# mTOR Overactivation in Mesenchymal cells Aggravates CCl_4_− Induced liver Fibrosis

**DOI:** 10.1038/srep36037

**Published:** 2016-11-07

**Authors:** Lanlan Shan, Yan Ding, You Fu, Ling Zhou, Xiaoying Dong, Shunzhi Chen, Hongyuan Wu, Wenqing Nai, Hang Zheng, Wanfu Xu, Xiaochun Bai, Chunhong Jia, Meng Dai

**Affiliations:** 1 Department of Health Management, Nanfang Hospital, Southern Medical University, Guangzhou, Guangdong, 510515, China; 2 Department of Oncology, Nanfang Hospital, Southern Medical University, Guangzhou, Guangdong, 510515, China; 3 Department of Gastroenterology, Guangzhou Women and Children’s Medical Center, Guangzhou Medical University, Guangzhou, 510623, China; 4 Department of Cell Biology, School of Basic Medical Sciences, Southern Medical University, Guangzhou, Guangdong, 510515, China

## Abstract

Hepatic stellate cells are of mesenchymal cell type located in the space of Disse. Upon liver injury, HSCs transactivate into myofibroblasts with increase in expression of fibrillar collagen, especially collagen I and III, leading to liver fibrosis. Previous studies have shown mTOR signaling is activated during liver fibrosis. However, there is no direct evidence *in vivo*. The aim of this study is to examine the effects of conditional deletion of *TSC1* in mesenchymal on pathogenesis of liver fibrosis. Crossing mice bearing the floxed *TSC1* gene with mice harboring *Col1α2*-Cre-ER(T) successfully generated progeny with a conditional knockout of *TSC1* (*TSC1* CKO) in collagen I expressing mesenchymal cells. *TSC1* CKO and WT mice were subjected to CCl_4_, oil or CCl_4_+ rapamycin treatment for 8 weeks. *TSC1* CKO mice developed pronounced liver fibrosis relative to WT mice, as examined by ALT, hydroxyproline, histopathology, and profibrogenic gene. Absence of *TSC1* in mesenchymal cells induced proliferation and prevented apoptosis in activated HSCs. However, there were no significant differences in oil-treated *TSC1* CKO and WT mice. Rapamycin, restored these phenotypic changes by preventing myofibroblasts proliferation and enhancing their apoptosis. These findings revealed mTOR overactivation in mesenchymal cells aggravates CCl_4_− induced liver fibrosis and the rapamycin prevent its occurance.

Liver fibrosis is a major cause of morbidity and mortality worldwide due to chronic viral hepatitis, alcoholic hepatitis, and nonalcoholic steatohepatitis. Fibrosis formation is a wound-healing response in response to liver injury that is characterized by the accumulation of extracellular matrix (ECM)^1^. Myofibroblasts (MF) are defined primarily by their ability to produce ECM and contractile activity^2^. Hepatic stellate cells (HSCs) were found to be the major source of myofibroblasts in a mouse model of CCl_4_− induced liver fibrosis^3^. The transformation of quiescent vitamin A-rich HSCs into proliferative, contractile, fibrogenic myofibroblasts following liver injury has launched an era of astonishing progress in understanding the mechanistic basis of hepatic fibrosis progression and regression. Moreover, this simple paradigm has yielded a remarkably broad appreciation of myofibroblast function, not only in liver injury, but also hepatic development, regeneration, xenobiotic responses, intermediary metabolism, and immunoregulation^4^. At the same time, countless studies have used tissue immunohistochemistry to identify HSCs, while the most prominent proteins analyzed to date include α-smooth muscle actin (α-SMA), desmin, and glial fibrillary acidic protein (GFAP)^5^.

The mammalian target of rapamycin (mTOR) nucleates two distinct multi-protein complexes, mTOR complex 1 (mTORC1) and mTOR complex 2 (mTORC2). mTORC1 has five components that include mTOR, regulatory-associated protein of mTOR (RAPTOR), target of rapamycin complex subunit LST8 (mLST8), proline-rich AKT1 substrate 1 (PRAS40), and DEP domain-containing mTOR-interacting protein (DEPTOR), while mTORC2 has six components that include mTOR, RPTOR-independent companion of mTOR (RICTOR), target of rapamycin complex 2 subunit MAPKAP1 (mSIN1), protein observed with Rictor-1 (Protor-1), mLST8, and DEPTOR^6^. The tuberous sclerosis complex (TSC), which comprises TSC1 and TSC2, is involved in the negative regulation of mTORC1 activity^7^. Loss of TSC1 causes cells and tissues to display constitutive mTORC1 activation. In response to numerous intracellular and extracellular stimuli, mTORC1 phosphorylates eukaryotic initiation factor 4E-binding protein-1 (4E-BP1) and S6 kinase 1 (S6K1), which exerts an essential role in increasing translation of a subset of mRNAs and accelerating growth and proliferation^6^,^8^. Under certain circumstances, inhibition of mTOR activity by rapamycin accelerates apoptosis^9^,^10^. Induction of mutations of mTOR can contribute to apoptotic resistance and might contribute to cellular transformation^11^.

Activation of the mTOR pathway has been reported in several fibrotic diseases. The mTOR pathway plays an important role in cardiac fibrosis and rapamycin is a potential therapeutic treatment that can be used to attenuate cardiac fibrosis^12^. The rapamycin analogue SDZ RAD was shown to promote dramatic inhibitory effects on collagen accumulation in the lungs in a bleomycin model of pulmonary fibrosis^13^, thus suppression of mTOR may be a viable treatment for pulmonary fibrosis^14^. mTORC1 signaling promotes the activation of kidney fibroblasts and contributes to the development of interstitial fibrosis^15^. There is growing evidence to support the idea that the mTOR signaling pathway plays a key role in liver fibrosis^16^. Activation of HSCs is regarded as a critical step in the pathogenesis of liver fibrosis^3^. HSCs are a resident mesenchymal cell type located in the subendothelial space of Disse that are interposed between the sinusoidal endothelium and hepatocytes^4^. Based on the findings of these studies, we speculate that activation of mTOR signaling in mesenchymal cells might also play a role in liver fibrosis.

To date, the specific effect of mTOR on liver fibrosis has not yet been reported. Therefore, the aim of the present study was to investigate the function of mTOR overactivation in mesenchymal cells in CCl_4_− induced liver fibrosis and to reveal possible mechanisms underlying its participation in fibrotic diseases.

## Results

### Generation of TSC1–conditional knockout mice in the mesenchymal compartment

**Figure 1:**
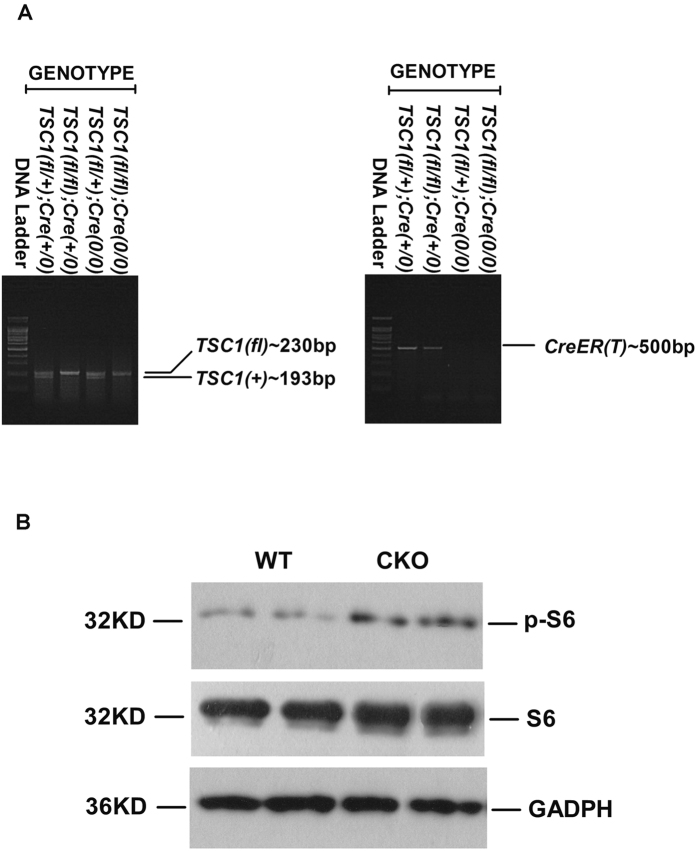
Generation of *TSC1* conditional knockout (CKO) mice in the mesenchymal compartment. Generation of *TSC1* conditional knockout (CKO) mice in the mesenchymal compartment **A**: Mouse genotype was detected by PCR. Genomic DNA samples from the indicated strains of mice were analyzed using primers specific for wild-type (WT) and floxed (fl) *TSC1*, or Cre-ER (T) mice. The PCR products and 100-bp DNA ladder were separated by agarose gel electrophoresis. The bands corresponding to the WT (193 bp) and fl (230 bp) TSC1 alleles are indicated, and confirm the correct *TSC1* genotype for each strain. The correct genotype with respect to Cre-ER(T) (500 bp) gene is confirmed in the respective strain. **B**: *TSC1* knockout efficiency was detected by western blotting after daily injection of tamoxifen for 8 days. Liver tissue samples from WT and *TSC1* CKO mice were analyzed for p-S6 (s235/236). The phosphorylation status of S6 was elevated, indicating TSC1 knock-down in the livers of *TSC1* CKO mice. S6 and GAPDH were included as loading controls.

Homozygous floxed *TSC1* mice (*TSC1*^*fl/fl*^) were cross-bred with mice harboring a *Cre-ER(T)* recombinase gene to generate Cre/*TSC1* heterozygous mice. The second cross generated [*TSC1*^*fl/fl*^*, Col1α2-CreER(T)*+/*0*] mice. Genomic DNA from tail tissue samples of these mice was genotyped by PCR analysis to confirm the presence of the *TSC1* and *Cre* genes (Fig. 1A). To delete the *TSC1* gene in the mesenchymal compartments, mice with genotype [*TSC1*^*fll/fl*^*, Col1α2-CreER(T)*+/*0*] were treated with tamoxifen as described in the Materials and Methods section and were referred to as *TSC1* CKO mice. Mice with genotype [*TSC1*^*fl/fl*^*,Col1α2-CreER(T)0*/*0*] under the same treatment were referred to as WT mice. Deficiency of TSC1 expression in the mesenchymal compartment was verified by western blot and immunofluorescence analyses. As shown in Figs 1B and 5B, expression levels of p-S6 (s235/236), mTOR, and the S6K1 downstream effector protein were strongly increased in *TSC1* CKO mice.

### TSC1 deletion augments CCl_4_− induced liver fibrosis in mice

**Figure 2:**
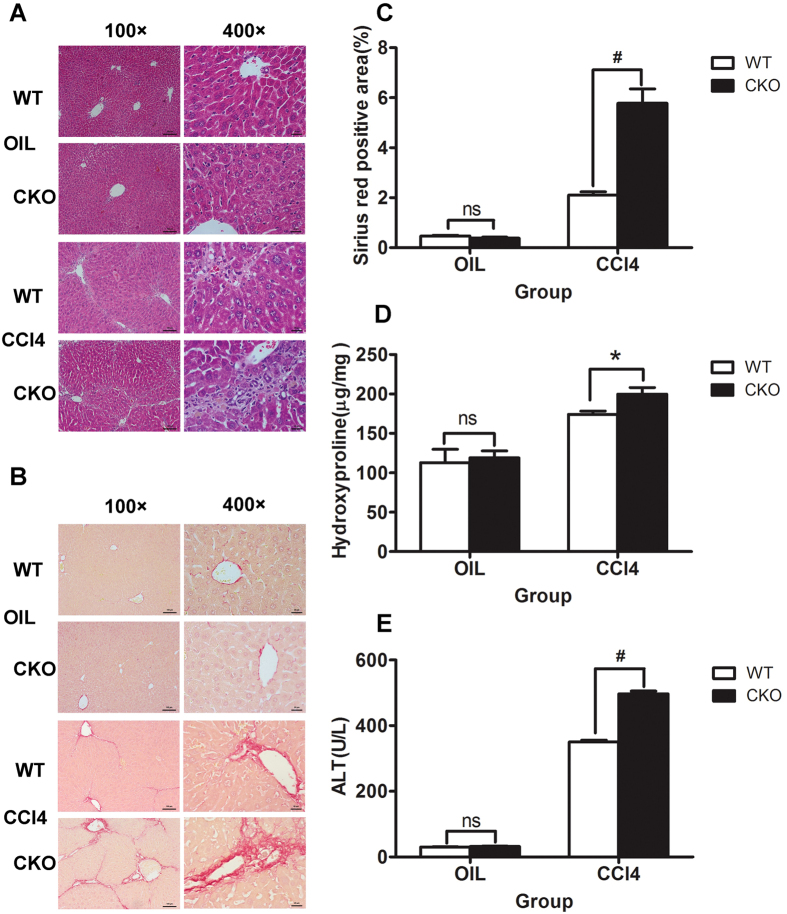
*TSC1* deletion augments CCl_4_− induced liver fibrosis in mice. Tamoxifen-treated control [wild-type (WT)] and *TSC1* conditional knockout (CKO) mice received intraperitoneal injections of either CCl_4_ or oil, as indicated. H&E staining (**A**) and Sirius Red staining (**B**) were performed 8 weeks later. *TSC1* CKO mice had more severe liver fibrosis than WT mice. All were imaged at 100x or 400x magnification. (**C**) The Sirius Red staining area in the liver was calculated by Image-Pro Plus 6.0. in six different images taken at 100x magnification on each slide. (**D**) The liver samples were analyzed for hydroxyproline content. (**E**) Liver injury was determined by serum ALT in WT and *TSC1* CKO mice. The results are presented as the mean ± SEM derived from three independent experiments. NS, *p* > 0.05; *p < 0.05; ^#^*p* < 0.01 for *TSC1* CKO vs. WT mice.

To evaluate the effect of *TSC1* deficiency in mesenchymal cells on liver fibrosis *in vivo*, *TSC1* CKO and WT mice were treated with CCl_4_, and the fibrotic response was evaluated. A histologic examination of liver sections using H&E and Sirius Red staining was performed, which showed that *TSC1* CKO mice exhibited greater degrees of lobular architecture damage and severe bridging necrosis than WT mice (Fig. 2A). *TSC1* CKO mice presented exacerbated fibrosis by the morphometric assessment of the total area positively stained with Sirius Red (Fig. 2B,C) and by higher liver hydroxyproline(HYP) content (174.09 ± 4.12 μg/mg vs. 199.78 ± 8.41 μg/mg, P < 0.05; Fig. 2D). Meanwhile, *TSC1* CKO mice exhibited higher serum ALT concentrations relative to WT mice (350.00 ± 5.77 U/L vs. 496.67 ± 8.82 U/L, P < 0.01; Fig. 2E). In comparison, there were no apparent differences in HYP (112.94 ± 9.74 μg/mg vs. 118.92 ± 5.07 μg/mg, P = 0.62) and ALT (30.00 ± 1.00 U/L vs. 31.67 ± 1.45 U/L, P = 0.40) detected after oil treatment between WT and *TSC1* CKO mice. Collectively, these results demonstrated that *TSC1* deletion in the mesenchymal compartment augments CCl_4_− induced liver fibrosis in mice.

### Increased α-SMA expression due to loss of TSC1

**Figure 3:**
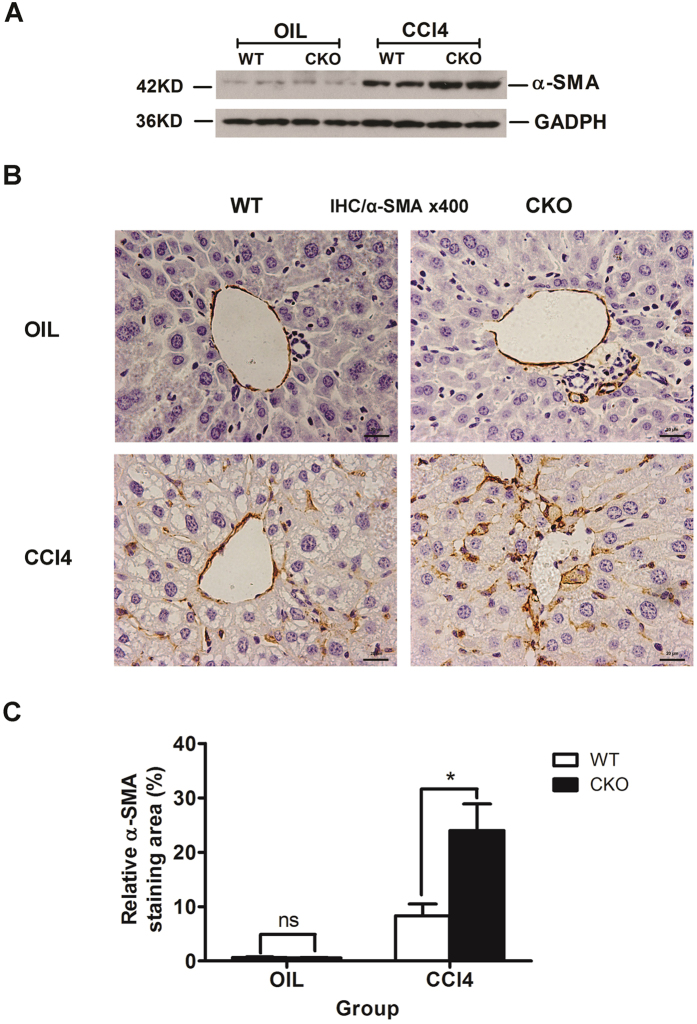
Increased α-SMA expression due to loss of *TSC1*. (**A**) Liver tissue samples from oil- or CCl_4_− treated mice for 8 weeks were analyzed for α-SMA protein expression by western blot analysis. The expression of α-SMA was significantly increased in CCl_4_− treated *TSC1* CKO mice. (**B**) IHC staining was performed with α-SMA in oil- and CCl_4_− treated mouse liver tissues. (**C**) α-SMA staining area in the liver was calculated by Image-Pro Plus 6.0. in six different images taken at 400x magnification on each slide. NS, *p* > 0.05; *p < 0.05 for *TSC1* CKO vs. WT mice.

The results of the above analyses showed that loss of *TSC1* resulted in exacerbation in CCl_4_− induced hepatic fibrosis. Consistent with enhanced liver fibrosis and collagen production, α-SMA positive staining was increased in CCl_4_− treated *TSC1* CKO mice (Fig. 3B,C). Western blot analysis confirmed significant CCl_4_− induced increases in α-SMA protein expression in *TSC1* CKO mice (Fig. 3A). However, there were no apparent differences in α-SMA expression detected after oil treatment between WT and *TSC1* CKO mice. These results suggest that *TSC1* deletion from mesenchymal cells promotes the development of liver fibrosis owing to the increasing number of α-SMA positive cells.

### Increased expression of profibrogenic markers in TSC1 CKO mice

**Figure 4:**
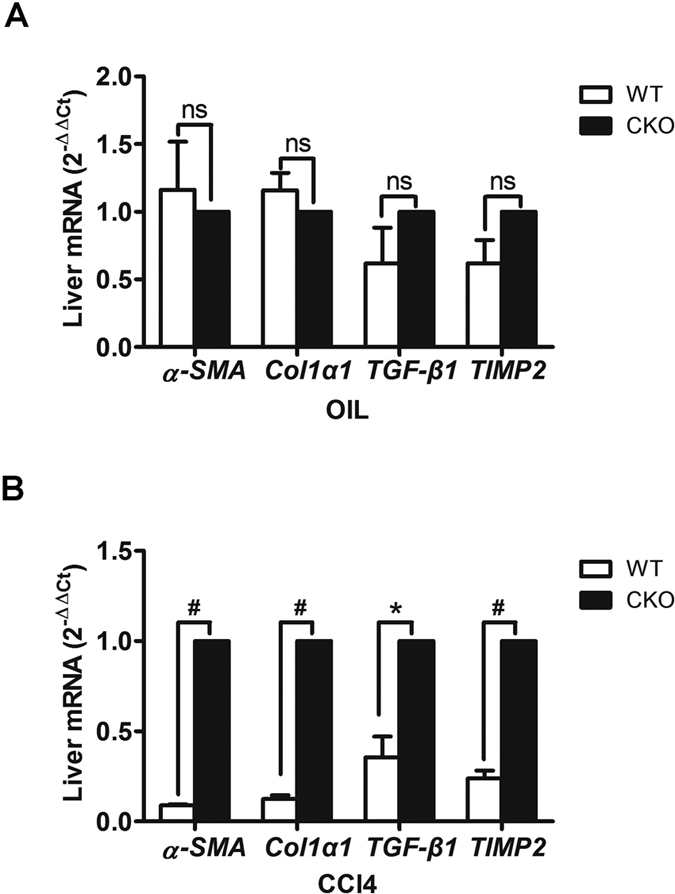
Increased expression of profibrogenic markers in *TSC1* CKO mice. (**A**) Expression levels of *α-SMA*, *Col1α1*, *TGF-β1*, and *TIMP2* were assessed by RT-PCR 8 weeks after treatment with oil. (**B**) Expression of *α-SMA*, *Col1α1*, *TGF-β1*, and *TIMP2* were assessed by RT-PCR 8 weeks after treatment with CCl_4_. Expression was normalized to the levels of *GAPDH* and results are presented as the mean ±  SEM derived from three independent experiments. NS, *p* > 0.05; *p < 0.05; ^#^*p* < 0.01 for *TSC1* CKO vs. WT mice.

RT-PCR was performed to quantify the expression of profibrogenic markers. As shown in Fig. 4A, levels of the profibrogenic markers *α-SMA*, *Col1α1*, *TGF-β1*, and *TIMP2* were unchanged between the two genotypes following treatment with oil. In CCl_4_− induced animals, there was a trend towards increased levels of *α-SMA*(11.2-fold; P < 0.01), *Col1α1* (8.3-fold; P < 0.01), *TGF-β1*(2.8-fold; P = 0.03), and *TIMP2*(4.2-fold; P < 0.01) in TSC1 CKO mice, as compared with WT mice (Fig. 4B). These results strengthened the evidence for the vulnerability of *TSC1* CKO mice to CCl_4_− induced fibrosis.

### Increased myofibroblasts in TSC1 CKO mice

**Figure 5:**
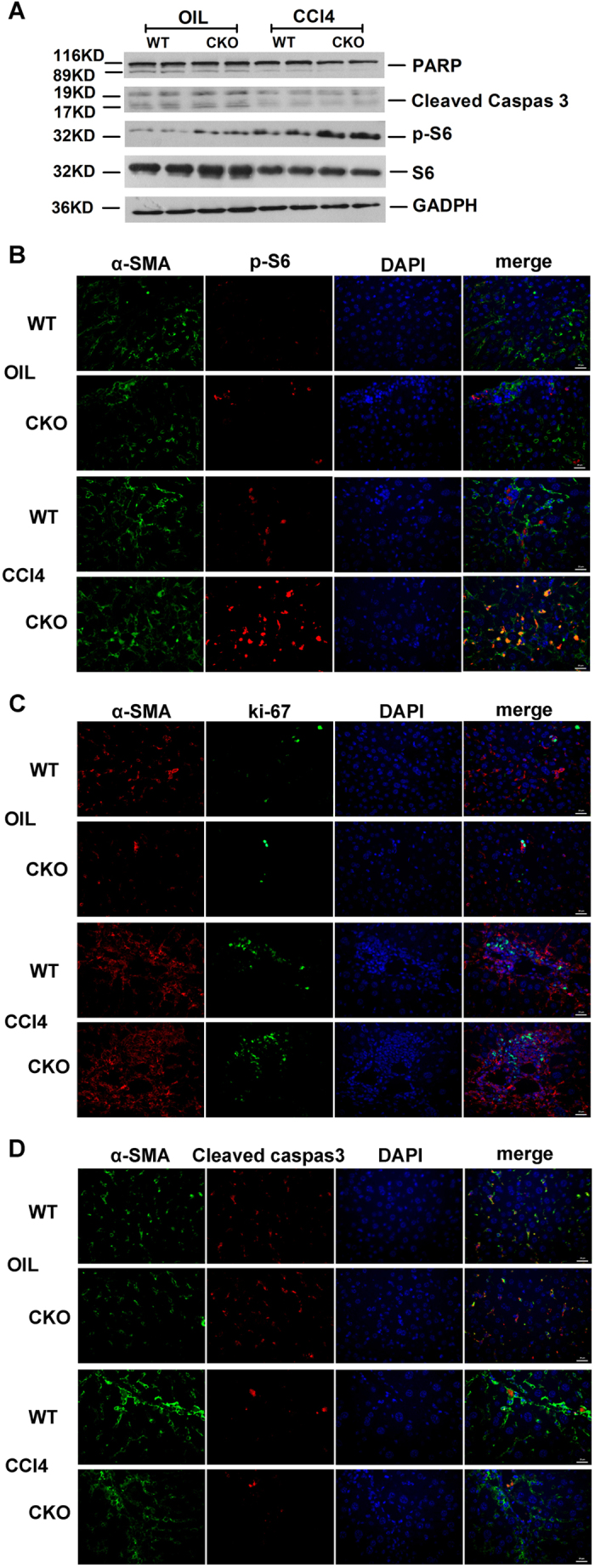
Increased myofibroblasts in *TSC1* CKO Mice. (**A**) Liver tissue samples from oil- or CCl_4_− treated mice were analyzed for PARP, cleaved caspase-3, p-S6, and S6 protein by western blot analysis. Immunofluorescence (IF) staining were performed to evaluate the impact of *TSC1* deletion on myofibroblasts proliferation and apoptosis. (**B**) Sections were stained with α-SMA (green) and p-S6 (red) antibodies. There were increased both α-SMA and pS6 positive cells in *TSC1* CKO mice administered CCl_4_. (**C**) Sections were stained with α-SMA (red) and ki67 (green) antibodies. Both α-SMA- and ki67-positive cells were increased in *TSC1* CKO mice following CCl_4_ treatment. (**D**) Sections were stained with α-SMA (green) and cleaved caspase-3 (red) antibodies. Both α-SMA- and cleaved caspase-3-positive cells decreased in the CCl_4_− treated *TSC1* CKO mice. Tissues were counterstained with DAPI (blue) to detect nuclei and imaged at 400x magnification.

To further confirm the importance of mTOR activity in myofibroblast proliferation in CCl_4_− induced liver fibrosis, protein expression was monitored by western blot and immunofluorescence analyses. As expected, p-S6 (s235/236) expression was upregulated in myofibroblasts from CCl_4_− treated mice, but was higher in the *TSC1* CKO mice than WT mice (Fig. 5A). Further detection of apoptotic proteins showed that expression levels of the cleaved form of caspase-3 and PARP were reduced in CCl_4_− treated mice, but was lower in the *TSC1* CKO mice than the WT mice (Fig. 5A). Myofibroblast formation was measured by immunofluorescence analysis with α-SMA antibody. Consistent with the western blotting results, increased phosphorylation of S6 with CCl_4_ treatment, at least partially, caused the number of myofibroblasts to increase in *TSC1* CKO mice (Fig. 5B). Following CCl_4_ treatment, ki67 and α-SMA double-positive cells, which are recognized as proliferative myofibroblasts, were significantly increased to a greater extent in *TSC1* CKO mice than in WT mice (Fig. 5C). Meanwhile, the abundance of cleaved caspase-3 and α-SMA double-positive cells, which are recognized as apoptotic myofibroblasts, was decreased in *TSC1* CKO mice, as compared with WT mice (Fig. 5D). Intriguingly, expression of α-SMA, ki67, and cleaved caspase-3 was not appreciably altered in oil group mice, indicating that under quiescent conditions, loss of *TSC1* in cells expressing collagen type I was not accompanied by an increase in the abundance of myofibroblasts in the liver.

### Rapamycin attenuates CCl_4_− Induced Liver Fibrosis and reverses phenotypes in TSC1 CKO mice

**Figure 6:**
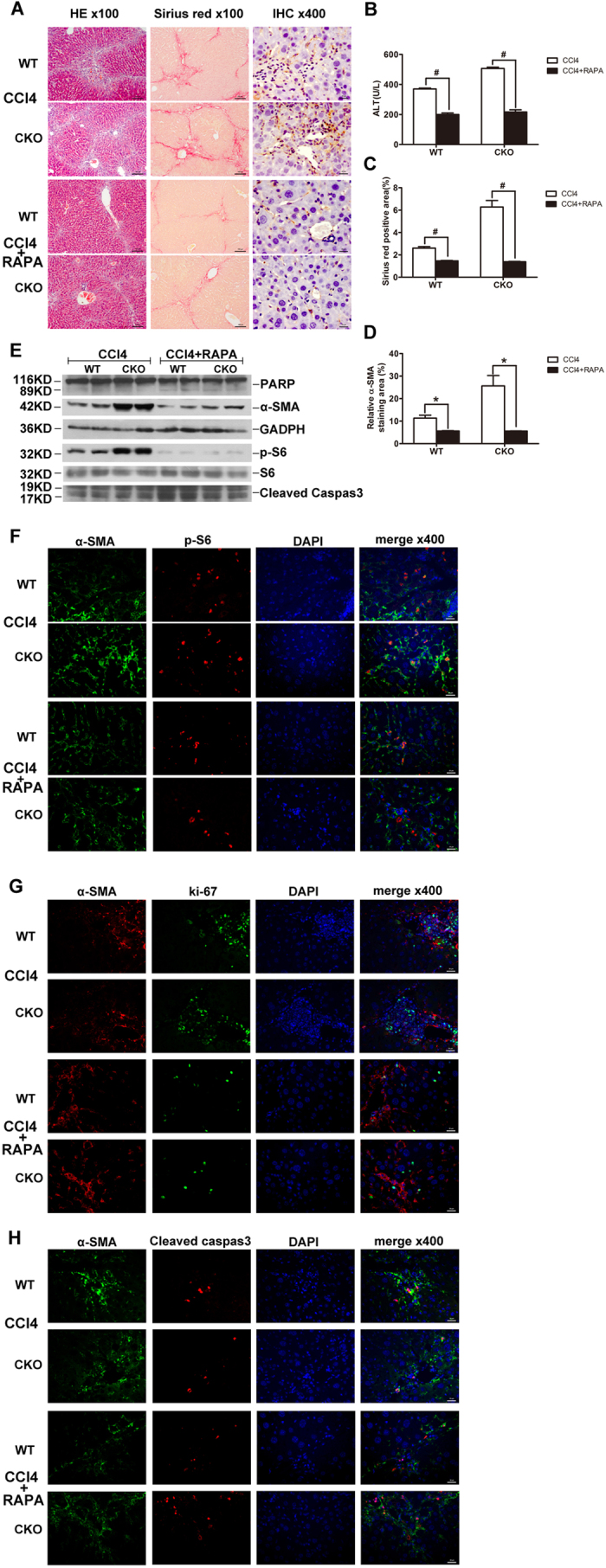
Rapamycin attenuates CCl_4_− Induced Liver Fibrosis and reverses phenotypes in TSC1 CKO mice (**A**) Liver tissue samples from CCl_4_− or CCl_4_+ rapamycin- treated mice for 8 weeks were analyzed for histological analysis. As shown in figure H&E staining and Sirius Red staining, CCl_4_+ rapamycin- treated mice had less severe liver fibrosis than CCl_4_− treated mice. All were imaged at 100x magnification. IHC staining was performed with α-SMA in CCl_4_− or CCl_4_+ rapamycin- treated mice liver tissues. (**B**) Liver injury was determined by serum ALT in CCl_4_− and CCl_4_+ papamycin-treated mice. (**C**) The Sirius Red staining area in the liver was calculated by Image-Pro Plus 6.0. in six different images taken at 100x magnification on each slide. (**D**) a-SMA staining area in the liver was calculated by Image-Pro Plus 6.0. in six different images taken at 400x magnification on each slide. (**E**) Liver tissue samples from CCl_4_− or CCl_4_+ rapamycin- treated mice were analyzed for PARP, cleaved caspase-3, p-S6, and S6 protein by western blot analysis. Immunofluorescence(IF) staining were performed to evaluate the impact of rapamycin on myofibroblasts proliferation and apoptosis. (**F**) Sections were stained with α-SMA (green) and p-S6 (red) antibodies. There were decreased α-SMA and pS6 double positive cells in mice administered CCl_4_+ rapamycin. (**G**) Sections were stained with α-SMA (red) and ki67 (green) antibodies.α-SMA- and ki67 double positive cells were decreased in mice following CCl_4_+ rapamycin treatment. (**H**) Sections were stained with α-SMA (green) and cleaved caspase-3 (red) antibodies. α-SMA- and cleaved caspase-3 double positive cells increased in the CCl_4_+ rapamycin-treated mice. Tissues were counterstained with DAPI (blue) to detect nuclei and imaged at 400x magnification. ^**#**^p < 0.01; *p < 0.05 for CCl_4_− vs. CCl_4_+ rapamycin-treated mice.

It is known from the literature that the rapamycin has inhibitory effects on liver fibrosis^17^, and this is consistent with our results. In CCl_4_+ rapamycin-treated CKO or WT mice, there was less severe liver histology injury and lower serum ALT concentrations compared with the mice merely treated with CCl_4_ (Fig. 6A,B). CCl_4_+ rapamycin-treated mice presented attenuated fibrosis by the morphometric assessment of the total area positively stained with Sirius Red (Fig. 6A,C). Immunohistochemical staining for α-SMA showed that α-SMA-positive staining was decreased in CCl_4_+ rapamycin-treated mice (Fig. 6A,D). As shown in Fig. 6E, western blot analyses indicated that α-SMA and p-S6 protein expression were lower in the CCl_4_+ rapamycin-treated mice in comparison with CCl_4_− treated mice, but the expression levels of the cleaved form of caspase-3 and PARP were upregulated in CCl_4_+ rapamycin-treated mice. Consistent with the western blotting results, decreased phosphorylation of S6 with rapamycin treatment, at least partially, caused the number of myofibroblasts to decrease (Fig. 6F). Ki67 and α-SMA double-positive cells were also significantly decreased to a greater extent in CCl_4_+ rapamycin-treated mice (Fig. 6G). Meanwhile, the number of cleaved caspase-3 and α-SMA double-positive cells were increased in CCl_4_+ rapamycin-treated mice, as compared with CCl_4_− treated mice (Fig. 6H). Intriguingly, there were no significant differences between the WT and the *TSC1* CKO in CCl_4_+ rapamycin-treated mice.

Thus, *TSC1* deficiency in the mesenchymal compartment resulted in a significant increase in the abundance of myofibroblasts, thereby contributing to the noted exacerbation of liver fibrosis. Conversely, rapamycin can alleviated CCl_4_− induced liver fibrosis by inhibiting myofibroblast proliferation and inducing apoptosis, and rescued the specific phenomenon by *TSC1* deficiency in the mesenchymal compartment.

## Discussion

Fibrosis is a pathological process characterized by excessive accumulation of connective tissue components in organs and tissues. Liver fibrosis develops as a consequence of liver injury and activation of HSCs, which represents the imbalance between ECM production and degradation. Previous studies have reported that mTOR was involved in the process of liver fibrosis in human tissues and animal models of CCl_4_− mediated fibrosis^18^,^19^. Understanding the importance of mTOR signaling in different cell compartments is critical for future studies to develop effective drugs targeting this signaling pathway with minimal off-target or negative adverse effects. In this study, the role of aberrant mTOR activity in the mesenchymal compartment in the pathogenesis of liver fibrosis in a conditional *TSC1* knockout mouse model was investigated, which showed that *TSC1* deletion in mesenchymal cells aggravated liver fibrosis and was associated with myofibroblast proliferation and apoptosis.

Growing evidence supports the hypothesis that mTOR overactivation is involved in the pathogenesis of fibrotic diseases, including liver fibrosis^16^,^20^,^21^,^22^. We specifically focused on the role of mesenchymal mTOR overactivation in liver fibrosis. Our data further indicated that the mTOR signaling pathway was activated in CCl_4_− mediated liver fibrosis (Fig. 5A,B), which is consistent with a previous study that reported mTOR activation in liver fibrosis in an animal model^19^. After obtaining evidence supporting our hypothesis that mTOR overactivation was involved in the process of liver fibrosis, we further demonstrated that mTOR activation resulting in increased myofibroblast accumulation in the liver could be a major mechanism underlying fibrosis formation.

When the Cre-ER(T) recombinase was activated by tamoxifen, TSC1 expression was selectively absent only in mesenchymal cells. HSCs, first described by Kupffer in the 19th century, have emerged in the past 25 years as remarkably versatile mesenchymal cells^4^. The mTOR signaling pathway may be activated in HSCs in *TSC1* CKO mice (Fig. 1C). As previously reported using other methodologies, HSCs are the major source of myofibroblasts (>87%) in CCl_4_− induced liver injury^23^.

Myofibroblasts are the major source of fibrogenic cytokines and ECM^24^. Increasing myofibroblast accumulation in *TSC1* CKO mice in response to CCl_4_ treatment is thought to contribute to the noted significant increase in liver fibrosis. This result is also consistent with a previous study in which rapamycin appeared to inhibit proliferation and differentiation of corneal myofibroblasts *in vitro*^25^.

mTOR regulates renal myofibroblast activity and proliferation, and also plays an important role in preventing apoptosis of activated HSCs and promoting progression of fibrosis^26^. Apoptosis of HSCs may be involved in the termination of this response^27^. In a microarray study of human HSCs, cells became senescent and switched from a primarily fibrogenic to an inflammatory phenotype, with decreased proliferation and gene expression, and increased apoptosis^28^. PI3K/AKT as the upstream of the mTOR signaling pathway, inhibition of PI3K/Akt signaling pathway has been shown to induce HSCs apoptosis and attenuate liver fibrosis *in vitro*^29^. These findings suggest that reduced myofibroblast proliferation and increased myofibroblast apoptosis could effectively reduce fibrosis.

Interestingly, the CCl_4_− treated *TSC1* CKO mice exhibited obvious differences in liver fibrogenesis relative to control mice. However, there were no significant differences in the oil group between *TSC1* CKO and WT mice. These results imply that the effect of mTOR pathway activation in mesenchymal cell in normal mice is balanced by tissue homeostasis. In the experimental hepatic fibrosis model, after liver cell injury, mTOR pathway activation in mesenchymal cell enhanced the wound healing response.

In summary, mTOR overactivation in the mesenchymal compartment augmented liver fibrosis induced by CCl_4_. The mechanism likely involved activation of the mTOR pathway, which induced myofibroblast proliferation and prevented apoptosis. Although these results confirmed the importance of mesenchymal mTOR activity on myofibroblast proliferation *in vivo*, additional and/or alternate mechanisms cannot be excluded, thus additional future studies are warranted. Inhibition of mesenchymal mTOR overactivation and the induction of myofibroblasts present potential treatments for liver fibrosis.

## Methods

### Mice

All animal experiments are approved by the Southern Medical University Animal Care and Use Committee. All animals received humane care and the study protocols were conducted in compliance with the institution’s guidelines. Mice importing, transporting, housing and breeding were conducted according to the recommendations of “The use of non-human primates in research.” *Col1α2CreERT* and *TSC1*^*fl/fl*^ mice were both purchased from The Jackson Laboratory (Bar Harbor, ME, USA). *Col1α2CreERT* mice express a tamoxifen-inducible Cre recombinase driven by the mouse Col1α2, collagen, type1, alpha 2, promoter. The transgene insert contains a fusion product involving Cre recombinase and a mutant form of the mouse estrogen receptor ligand binding domain. *Cre* (*C57Bl/6*) transgenic mice were crossed with mice having both *TSC1* alleles floxed (*TSC1*^*fl/fl*^) (*129S4/SvJae)* to generate mice heterozygous for both alleles. Mice heterozygous for both alleles were backcrossed with *TSC1*^*fl/fl*^ mice to yield *Col1α2CreERT*/*TSC1*^*fl/fl*^ mice.

To selectively delete *TSC1* in Cre expressing cells, *Col1α2CreERT* /*TSC1*^*fl/fl*^, mice (6–8 weeks old) received daily intraperitoneal injections of the tamoxifen suspension (0.1 ml of diluted stock) for 8 days (*TSC1* CKO), while TSC1 wild-type (WT) mice, as controls, received the same treatment. After administration of the tamoxifen treatment regimen, 8–12-week-old male *TSC1* CKO and WT mice were induced by intraperitoneal injection with 20%CCl_4_ (5 μl/g body weight; Sigma Chemicals, Heidelberg, Germany) dissolved in olive oil for 8 weeks. The control group received the same volume of olive oil only. In the CCl_4_+ rapamycin group, after treatment with CCl_4_, the mice also received rapamycin (1 mg/kg body weight/day; Sigma-Aldrich) by intraperitoneal injection daily until sacrifice.

### Hydroxyproline Assay

The hydroxyproline assay was used to estimate the percentage of degraded collagen^30^. To assess the extent of fibrosis, the collagen content of liver homogenates after alkaline hydrolysis was assayed using a colorimetric assay (Nanjing Jiancheng Bioengineering Institute, Nanjing, China).

### Alanine transaminase (ALT)

Serum was obtained by cardiac puncture from anesthetized mice following overnight fasting for detection of ALT levels using an IDEXX Catalyst Dx Biochemical Analyzer (IDEXX Laboratories, Westbrook, ME, USA).

### Histological analysis

For histological analysis, liver samples were fixed for 24 h in 4% paraformaldehyde, embedded in paraffin, sliced into 5 μm-thick sections, and stained with hematoxylin and eosin (H&E)or Sirius Red, as previously described^31^.

For immunohistochemical analysis, mouse liver sections were deparaffinized, rehydrated, and incubated with α-SMA antibody (1:100; Abcam, Cambridge, UK) overnight at 4 °C. The same concentration of normal mouse IgG served as a negative control. The bound antibodies were then visualized using diaminobenzidine as a chromogen and the slides were counterstained with hematoxylin. All sections were observed and photographed using an Olympus BX51 microscope (Olympus Corporation, Tokyo, Japan). The area of positive staining was measured in six different images taken at 400x magnification on each slide and quantified using Image Pro Plus 6.0 software (Media Cybernetics, Rockville, MD, USA).

For immunofluorescent staining, the sections were incubated with antibodies against phospho-S6 ribosomal protein (Ser235/236) (1:100; Cell Signaling Technology, Boston, MA, USA), α-SMA (1:100; Abcam), Ki67 (1:200; GeneTex, Inc., Irvine, CA, USA), and cleaved caspase-3 (1:200; Cell Signaling Technology). After incubation with the primary antibodies, the sections were then washed with phosphate-buffered saline and incubated with appropriate fluorescent secondary antibodies (1:200; Invitrogen Corporation, Carlsbad, CA, USA). Sections were mounted using 4′,6-diamidino-2-phenylindole (DAPI) and imaged by fluorescent microscopy.

### Western blot analysis

Protein extracts were separated by gel electrophoresis, transferred to nitrocellulose membranes, and subsequently incubated with antibodies against mouse phospho-S6 ribosomal protein (Ser235/236) (1:2000; Cell Signaling Technology), S6 (1:3000; Santa Cruz Biotechnology Inc., Dallas, TX, USA), α-SMA (1:3000; Abcam), PRAP (1:1000; Cell Signaling Technology), cleaved caspase-3 (1:2000; Cell Signaling Technology), or glyceraldehyde 3-phosphate dehydrogenase (1:3000; Santa Cruz Biotechnology Inc.). Thereafter, the blots were washed three times with TBST (50 mM Tris, 150 mM NaCl, 0.05% Tween 20, adjusted to pH 7.6 with HCl) for 5 min and then incubated with horseradish peroxidase-conjugated secondary antibodies, and visualized using an enhanced chemiluminescence kit (PerkinElmer, Inc., Waltham, MA, USA).

### Real-time polymerase chain reaction (RT-PCR) analysis

Total RNA was isolated using TRI reagent (Sigma Chemicals) and cDNA was generated using Primescript RT master mix (Takara Bio, Inc., Ōtsu, Japan). Duplicate PCR amplifications were carried out using a Light Cycler LC480 Real-Time PCR System (Roche, Basel, Switzerland) with the SYBR Green PCR Kit (Takara Bio, Inc.). mRNA concentrations were calculated using the 2^−ΔΔCt^ method. The primers used in this study were produced by Sangon Biotech Co., Ltd. (Shangai, China) and the sequences are listed in Table 1.

### Statistical analysis

All data are expressed as the mean ± standard error of the mean (SEM) from three individual experiments. The data for each group were analyzed using the *t*-test with SPSS ver. 20.0 software (IBM-SPSS, Inc., Chicago, IL, USA). A probability (*p*) value of < 0.05 was considered statistically significant.

## Additional Information

**How to cite this article**: Lanlan, S. *et al*. mTOR Overactivation in Mesenchymal cells Aggravates CCl_4_ − Induced liver Fibrosis. *Sci. Rep*. **6**, 36037; doi: 10.1038/srep36037 (2016).

**Publisher’s note**: Springer Nature remains neutral with regard to jurisdictional claims in published maps and institutional affiliations.

## Figures and Tables

**Table 1:** Primer sequences and accession numbers for primers used for RT-PCR.

Gene	Accession No.	Forward Primer (5′-3′)	Reverse Primer (5′-3′)
*α-SMA*	NM_007392.4	GTCCCAGACATCAGGGAGTAA	TCGGATACTTCAGCGTCAGGA
*col1α1*	NM_007742.3	GCTCCTCTTAGGGGCCACT	CCACGTCTCACCATTGGGG
*TGF-β1*	NM_011577.4	CTCCCGTGGCTTCTAGTGC	GCCTTAGTTTGGACAGGATCTG
*TIMP2*	NM_011594.1	TCAGAGCCAAAGCAGTGAGC	GCCGTGTAGATAAACTCGATGTC
